# A Case of Thromboembolic Cerebral Infarction Occurring Perioperatively in Acute Lower Limb Embolic Ischemia

**DOI:** 10.7759/cureus.106055

**Published:** 2026-03-29

**Authors:** Kanako Takai, Tsubasa Mikami, Akira Marumoto, Takashi Yamauchi, Minoru Ichikawa

**Affiliations:** 1 Department of Cardiovascular Surgery, Higashiosaka City Medical Center, Higashiosaka, JPN; 2 Department of Cardiology, Higashiosaka City Medical Center, Higashiosaka, JPN

**Keywords:** acute lower limb ischemia, anticoagulant therapy, cardioembolic infarction, cardioembolic stroke, embolic acute lower limb ischemia, thrombectomy, warfarin

## Abstract

Recurrent embolic events are not unusual in patients with non-valvular atrial fibrillation, and management of anticoagulation in these patients can be particularly challenging. We report a case of a patient who experienced multiple embolic events in the lower extremities and cerebral circulation despite ongoing anticoagulant therapy for atrial fibrillation. The patient was an 88-year-old woman with chronic kidney disease Stage G3b who was taking oral steroids for rheumatoid arthritis. She was also taking a direct Xa inhibitor (edoxaban) for chronic atrial fibrillation, with a CHADS_2_ score of 2 (age ≥75 years, and presence of hypertension) and a high CHA_2_DS_2_-VASc score of 4 (2 points for age ≥75 years, 1 point for hypertension, and 1 point for female sex, for a total of 4 points). The patient had been admitted to another hospital for calculous cholangitis and experienced symptomatic improvement after stone extraction and endoscopic sphincterotomy. During treatment for cholangitis, the patient required fasting and temporary discontinuation of edoxaban for only one day. On the day after resuming the direct oral anticoagulant following a one‑day interruption, she developed acute lower limb embolic ischemia and was referred to us. The patient underwent thrombectomy for an occluded superficial femoral artery. Following the restoration of blood flow, she developed cerebral embolism perioperatively while taking a different direct Xa inhibitor (rivaroxaban). She was subsequently switched to warfarin, and no further embolism has occurred for two years. In high‑risk cases of embolism, the use of warfarin, which permits precise dose adjustment, as the anticoagulant in the relatively early postoperative period may be a reasonable consideration, although this approach remains subject to debate.

## Introduction

Acute lower limb ischemia (ALI) arises from the two pathologies of atherosclerotic in situ thrombosis, acute on chronic limb ischemia, and embolism caused by intracardiac thrombi or mural thrombi in central arteries. In a previous report, of 73 cases with ALI, 35 were due to acute on chronic limb ischemia, and 38 cases were due to embolism [1].

A previous report [2] indicated that approximately 90% of embolic ALI cases occurring in individuals aged older than 50 years originated from intracardiac thrombi, whereas younger patients more commonly had proximal arterial thrombosis as the source of embolism. In particular, in elderly patients with atrial fibrillation (AF), cardiogenic embolism due to intracardiac thrombus should be strongly considered as a potential cause when ALI occurs.

Regarding the etiology of ischemic stroke, among patients aged over 50 years with ischemic stroke, 25.8% have been reported to have cardiogenic embolism as the underlying cause [3]. AF is observed in approximately 20%-30% of patients with ischemic stroke [4]. Therefore, it is recognized that cardiogenic embolism represents a significant and non-negligible cause of both cerebral and lower-extremity embolic events, and anticoagulant therapy for chronic AF is important. However, the rate of pre-event antithrombotic therapy in embolic ALI cases is relatively low at 44.7% [1], and a certain number of cases still develop embolism despite anticoagulant therapy.

We report a case of embolic stroke that occurred despite anticoagulant therapy for chronic AF after thrombectomy for ALI and discuss considerations regarding anticoagulation strategies in patients who experience recurrent cardioembolic events.

## Case presentation

**Figure 1:**
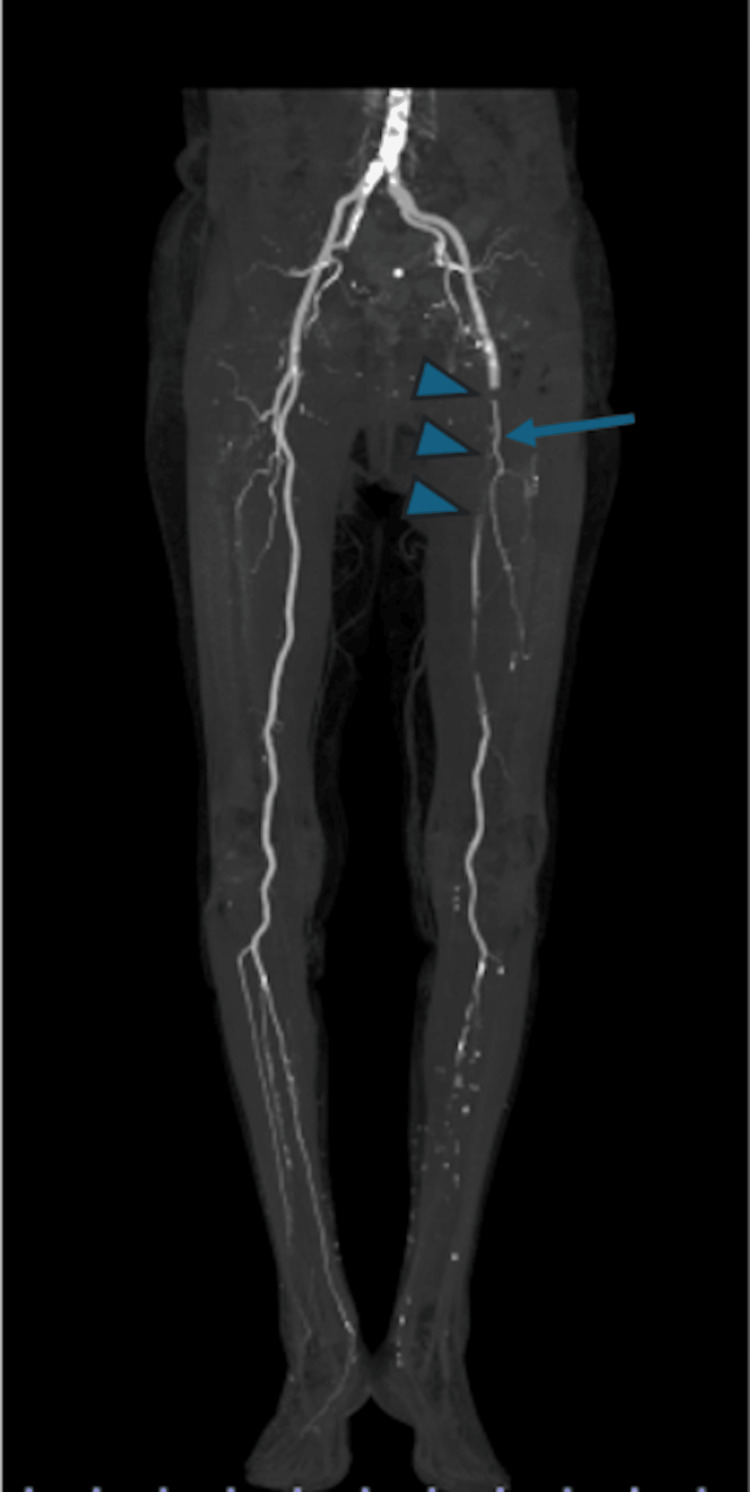
Preoperative contrast-enhanced CT angiography Contrast-enhanced computed tomography angiography shows obstruction of the left superficial femoral artery (SFA) from the ostium (arrow heads). Contrast enhancement was observed in the branches of the deep femoral artery (arrow). The collateral circulation of the SFA was poor.

An 88-year-old woman was hospitalized at another institution for calculous cholangitis. She had chronic AF and, with a body weight of 47.2 kg and a creatinine clearance (Ccr) of approximately 30 mL/min, fulfilled the criteria for dose reduction of edoxaban, for which she was receiving 30 mg daily. Although the exact duration of AF was unknown, AF had been documented on an electrocardiogram performed at least four years earlier. The CHADS_2_ score was 2 (age ≥75 years, and presence of hypertension), and the CHA_2_DS_2_-VASc score was 4 (2 points for age ≥75 years, 1 point for hypertension, and 1 point for female sex, for a total of 4 points). The patient had a HAS‑BLED score of 1, corresponding to a moderate bleeding risk. She was also taking prednisolone 5 mg orally for rheumatoid arthritis. Despite her super-elder age and a history of left hip arthroplasty, her ambulatory function had been preserved. Her cholangitis was improved with stone extraction and endoscopic sphincterotomy, and the interruption of edoxaban was limited to a single day (X-2), during which fasting was required. Edoxaban oral administration was resumed on day X-1, and the referring physician considered intravenous anticoagulation therapy unnecessary. On day X, she suddenly developed left lower limb pain and was transferred to our hospital with suspected lower limb arterial occlusion. Contrast-enhanced computed tomography (CT) showed occlusion of the left superficial femoral artery (SFA) (Figure 1). Based on the minimal atherosclerotic changes at this site, absence of collateral circulation, and presence of persistent AF, a diagnosis of acute lower extremity arterial occlusion due to cardiogenic embolism was deemed most appropriate. The patient was admitted urgently on the same day for thrombectomy. 

**Figure 2:**
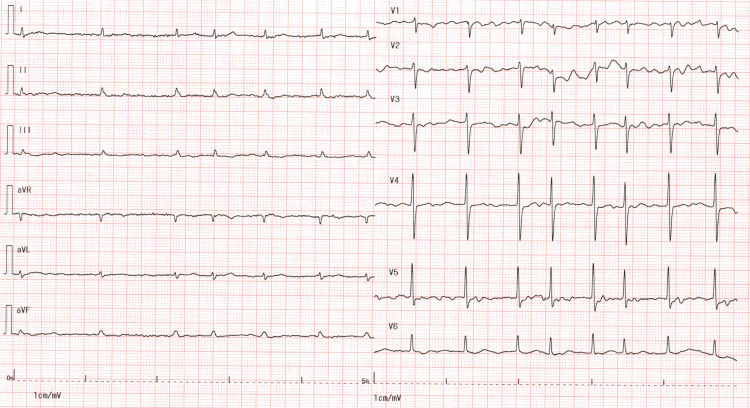
Electrocardiogram upon emergency admission The electrocardiogram on admission showed a heart rate of 92 beats per minute and demonstrated atrial fibrillation, characterized by the absence of P waves and irregular RR intervals.

**Figure 3:**
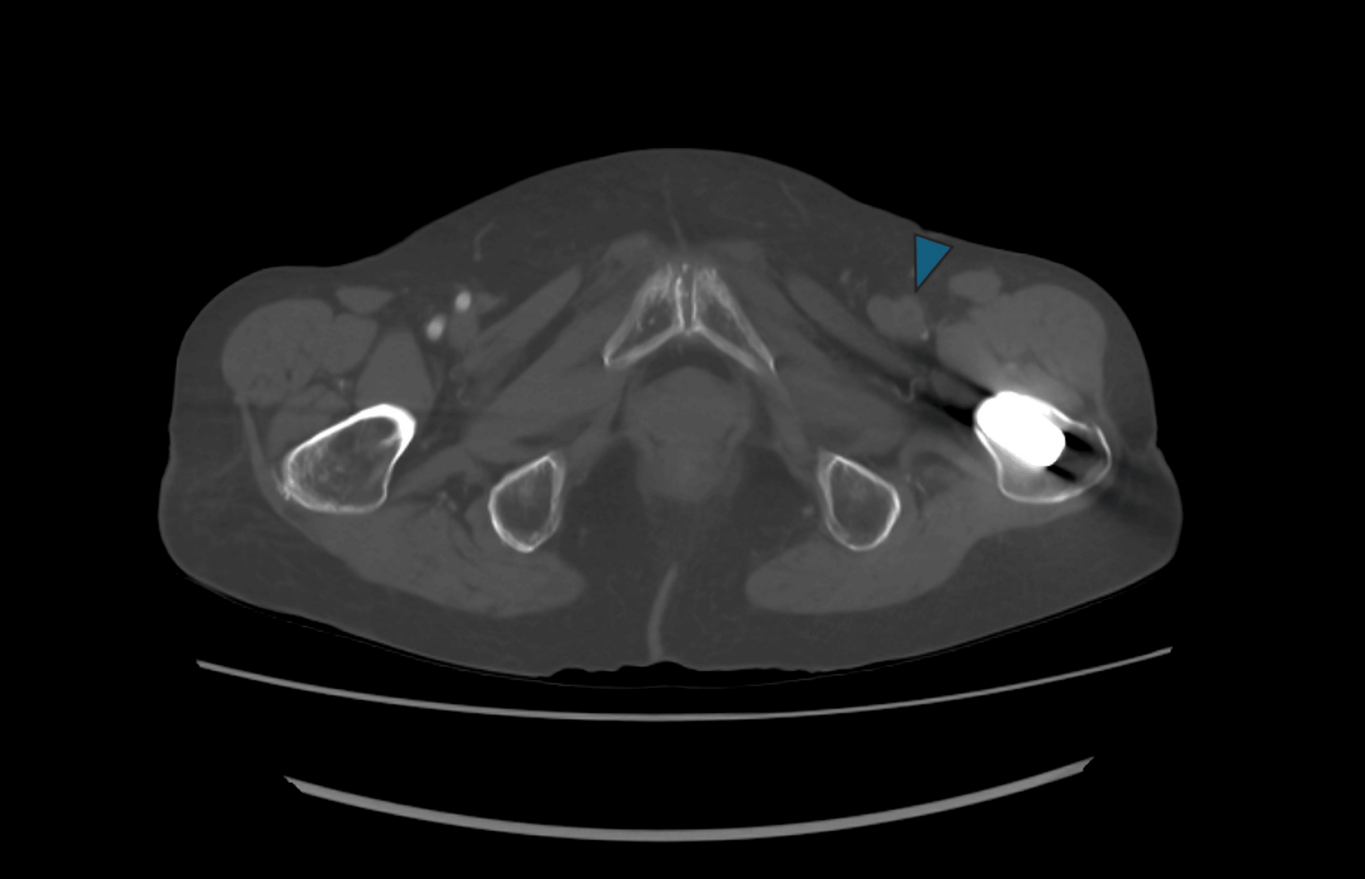
Preoperative contrast-enhanced CT angiography (axial view) Arterial wall calcification of the left femoral artery (arrowhead) was minimal. The CT attenuation value of the occluded vascular lumen was approximately 40 HU.

Rest pain and cyanosis were observed in the left lower leg. Palpation of the dorsalis pedis and posterior tibial arteries was not possible using Doppler ultrasound, and the Doppler-detected venous flow signal was also absent. Dorsiflexion and plantar flexion of the left ankle were possible, and no paralysis or sensory disturbance was found in the lower leg. An electrocardiogram upon admission showed a heart rate of 92 beats per minute and demonstrated AF, characterized by the absence of P waves and irregular RR intervals (Figure 2). Blood test results showed that the creatinine kinase concentration was not elevated (64 IU/L). The low-density lipoprotein level was 104 mg/dL, triglycerides were 90 mg/dL, and CRP was 4.08 mg/dL, indicating mild elevation. Serum creatinine was 0.98 mg/dL, the estimated glomerular filtration rate was 40.5 mL/min/1.73 m², and the Ccr calculated using the Cockcroft-Gault equation was 29.5 mL/min. A preoperative cardiac ultrasound performed nine months previously during an outpatient visit showed the following: ejection fraction, 61%; LVDd/Ds, 45/31 mm; LAD, 46 mm; LAVI, 52 mL/m²; AR, trivial; MR, grade, I; PR, severe; PRPG, 37 mmHg; and no evidence of thrombus in the cardiac chambers. Emergency contrast-enhanced CT showed occlusion from the origin of the left SFA to the mid-thigh. From the distal end of this occlusion to the popliteal artery, scattered thrombi were observed (Figure 1). The CT density of the occluded vessel lumen was 30-40 HU. Contrast enhancement was visible in the lower leg from the origin of the anterior tibial artery to approximately 14 mm beyond. Contrast enhancement was present up to the tibiofibular trunk but not beyond it. Although mild calcification was observed in the aorta, calcification was minimal in arteries distal to the femoral artery (Figure 3). Furthermore, no development of collateral circulation, which would suggest chronic occlusion, was observed (Figure 1). From the aortic arch to the abdominal aorta, there were no findings suggestive of irregular atherosclerotic lesions or ulcerative lesions. Furthermore, no obvious atherosclerotic lesions were observed from the contralateral iliac artery to the arteries of the foot.

**Figure 4:**
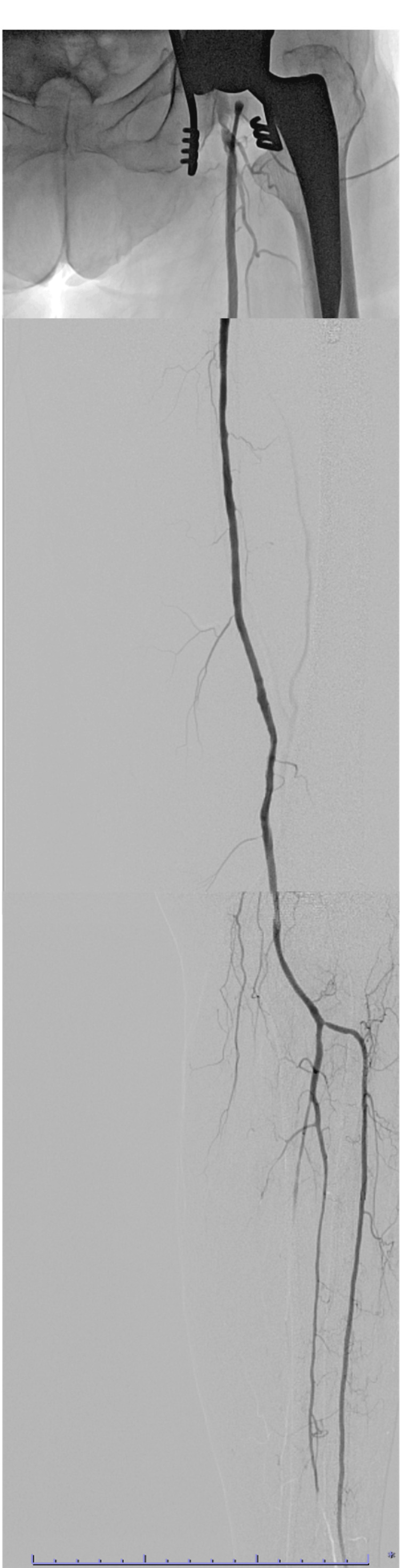
Intraoperative angiography Intraoperative angiography shows that the SFA to the lower leg was perfused without delay in a straight line up to the anterior tibial artery. Complete revascularization was achieved in at least one vessel extending to the lower leg. SFA, superficial femoral artery.

The patient was diagnosed with Rutherford class 2a acute lower extremity arterial occlusion and received 3000 units of heparin before undergoing thrombectomy under general anesthesia. The procedure commenced 7 hours after symptom onset and 2 hours and 40 minutes after hospital admission. Surgery was performed in the supine position under general anesthesia. A 4-cm longitudinal incision was made in the left groin. The common femoral artery, SFA, and deep femoral artery were exposed and taped. After administering 5000 units of heparin, the central common femoral artery was occluded with a bulldog clamp. A red thrombus was removed through the anterior half of the incision. Under fluoroscopy, a 0.025-inch guidewire was advanced into the SFA, and a 4 Fr Fogarty catheter (Edwards Lifesciences, Irvine, CA, USA) was used to remove a 10-cm long red thrombus. Contrast agent was injected into the distal SFA through the Fogarty catheter, and unobstructed opacification beyond the popliteal artery was confirmed. The catheter was then withdrawn proximally to confirm the absence of residual thrombus in the SFA. After suturing the common femoral artery closed, a purse-string suture was placed on the SFA, and a 6 Fr sheath was inserted. Contrast injection through the sheath confirmed uninterrupted, delay-free contrast flow in a straight line from the SFA to the distal tibial artery, and the procedure was finished. Intraoperative angiographic images are shown in Figure 4. As adequate opacification from the SFA to the anterior tibial artery was confirmed without any delay, we determined that adjunctive endovascular therapy was unnecessary and concluded the procedure with thrombectomy alone. The operative time was 72 minutes, with minimal intraoperative blood loss (gauze and suction).

Palpation of the dorsal foot artery was good postoperatively. Immediately postoperatively, the activated clotting time was prolonged at 376 seconds. The activated clotting time was shortened to 180 seconds at 9 hours postoperatively, which prompted the initiation of heparin at 5000 units/day. Anticoagulation therapy was changed from edoxaban 30 mg/day to rivaroxaban 10 mg/day starting the day after surgery. Renal function at this point was indicated by a Ccr of approximately 30 mL/min. A cardiac ultrasound performed on postoperative day 6 in the morning showed mild left atrial enlargement (45 mm), with no valvular disease or intracavitary thrombus. There were no new symptoms in the left lower extremity, and ambulation was unproblematic. At that time, the patient’s CHA_2_DS_2_‑VASc score was 6, with 2 additional points attributed to the history of embolic events.

On postoperative day 6 (hospital day 7), while preparing for a shower with a nurse after lunch, the patient suddenly developed impaired consciousness and paralysis of the right upper and lower extremities. Magnetic resonance imaging led to the diagnosis of cerebral infarction due to occlusion of the left middle cerebral artery. Emergency thrombectomy and intra-arterial heparin administration were performed, which resulted in improvement of the paralysis and impaired consciousness. Continuous intravenous heparin was initiated, rivaroxaban was discontinued, and oral warfarin potassium was started the following day.

The cerebral infarction was diagnosed as cardioembolic stroke because no stenosis was found in the middle cerebral artery or the origin of the internal carotid artery. Catheter ablation for AF was considered because of the recurrent cardioembolic events. Ultimately, the cardiologists determined that the patient, being super‑elderly and having long‑standing persistent asymptomatic AF, was not an appropriate candidate for the procedure. Transesophageal echocardiography for intracardiac thrombus was also considered unnecessary because of the decision against performing invasive procedures on older patients. Therefore, management was planned solely with warfarin anticoagulation therapy. Although the patient had no issues with activities of daily living and was fit for discharge home, she wished to continue rehabilitation and was transferred to another hospital on day X+27. Subsequently, on day X+55, the patient was discharged from the hospital. The patient continued taking warfarin orally until discharge from the rehabilitation hospital.

**Figure 5:**
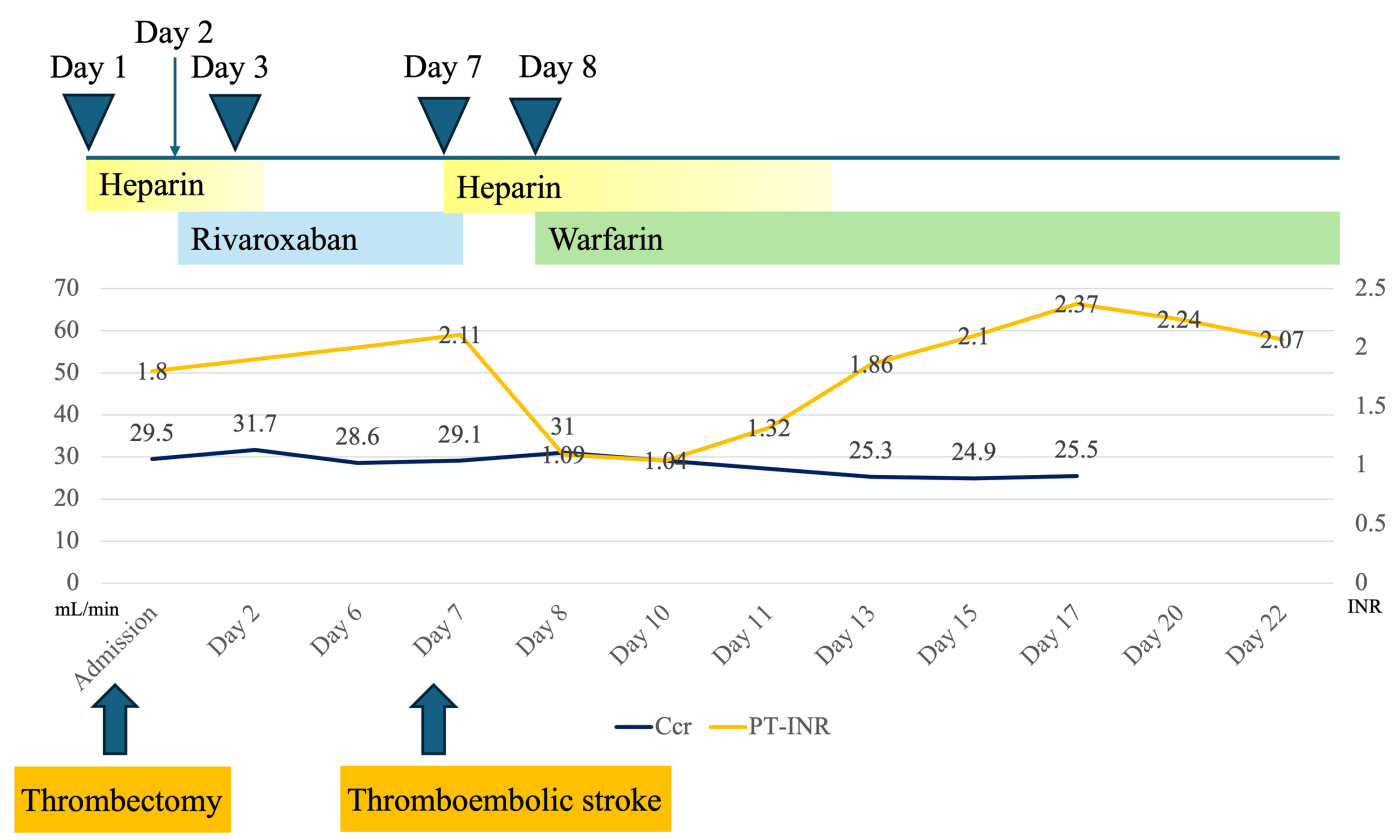
Transitive graph of the PT-INR and Ccr Heparin 5,000 units was initiated nine hours after surgery due to a shortened APTT, and rivaroxaban 10 mg was started the following day. After the onset of cerebral embolism, warfarin 1.5 mg was initiated in combination with heparin, and the dose was adjusted to maintain a target PT‑INR of 1.6-2.6. The Ccr value during the postoperative course remained at approximately 25-31 mL/min. APTT, activated partial thromboplastin time; Ccr, creatinine clearance; PT-INR, prothrombin time-international normalized ratio.

Figure 5 shows the type and duration of postoperative anticoagulant therapy, along with the progression of prothrombin time-international normalized ratio values and Ccr values. Two years have passed since thrombectomy, and there have been no subsequent thromboembolic events.

## Discussion

We report a case of a patient with non-valvular AF who developed a new cardiac cerebral embolism during the perioperative period of ALI caused by cardiogenic embolism.

The patient’s preoperative CHADS_2_ score was 2, and the CHA_2_DS_2_-VASc score was 4, which indicated a high-risk category. Oral anticoagulation was recommended according to the guidelines of the European Society of Cardiology [5]. We evaluated whether the anticoagulant therapy at that time was appropriate, given that the patient developed ALI despite being on edoxaban. Regarding the comparison between direct oral anticoagulants (DOACs) and warfarin, large-scale clinical trials have shown the non-inferiority of DOACs in preventing cerebral infarction and systemic embolism [6,7]. 

The ANAFIE Registry [8], which included patients aged 75 years or older with non-valvular AF, many of whom had high CHADS_2_ scores and elevated bleeding risks, demonstrated that DOACs were associated with lower rates of stroke and systemic embolism. Our patient was 88 years old and had chronic kidney disease Stage G3b or G4, which indicated that she had a relatively high bleeding risk. Therefore, selecting a DOAC as the preoperative anticoagulant was considered appropriate. Factors potentially contributing to thrombus formation before ALI onset include discontinuation of oral medication because of fasting associated with cholangitis, possible coagulation abnormalities induced by infection, and dehydration.

After thrombectomy for ALI, rivaroxaban was selected as the anticoagulant for our patient. This change in type of DOAC was made because ALI developed while the patient was receiving edoxaban. Despite rivaroxaban therapy, cerebral embolism occurred. However, taking into account renal function (Ccr: 30 mL/min), preoperative edoxaban 30 mg/day and postoperative rivaroxaban 10 mg/day dosages were consistent with current guideline recommendations and did not constitute off-label underdosing [9].

A previous study [10] reported that, in patients who develop ischemic stroke while taking DOACs, the annual incidence of recurrent embolic events or bleeding is as high as 13.4%. In particular, recurrent embolism has been associated with a high CHA_2_DS_2_‑VASc score at the time of the initial stroke and the presence of comorbid hypertension [10]. In this case, the patient’s CHA_2_DS_2_‑VASc score after the initial embolic event was 6, clearly indicating a high risk of recurrent embolic events and bleeding. Therefore, in addition to ensuring strict anticoagulation therapy after surgery, we performed at least a transthoracic echocardiogram to confirm the absence of thrombi within the cardiac chambers, including the left atrial appendage, that could serve as potential embolic sources.

In this case, a new cardiogenic embolism developed perioperatively during thrombectomy. A study that has evaluated the time to recurrent ischemic stroke indicated a median time of 34.2 days from the initial ischemic stroke to recurrent ischemic stroke [11], suggesting a tendency toward relatively early recurrence. Therefore, recurrence is likely to occur early after the initial event.

In addition, in this case, prednisolone administration for rheumatoid arthritis treatment may have increased the thrombotic risk. The patient’s history of rheumatoid rapid destructive hip arthritis also made discontinuation of prednisolone difficult, which might have contributed to the outcome. Although the mechanisms by which corticosteroids increase thrombotic risk are not fully elucidated, one report [12] demonstrated that healthy male subjects receiving oral dexamethasone exhibited significantly higher plasma levels of coagulation factors VII, VIII, XI, and fibrinogen than controls. Fibrinogen is a major determinant of plasma viscosity [13], and elevated levels are considered to contribute to hemodynamic disturbances, thereby fulfilling the “abnormal blood flow” component of Virchow’s triad. The risk of deep vein thrombosis associated with oral steroid use is well known [14], but no published studies have explicitly addressed the risk of intracardiac thrombus formation. However, theoretically, oral steroid use may also increase the risk of intracardiac thrombus formation, and it is possible that corticosteroid therapy contributed to thrombus development in our case.

This case involved a high risk of embolism. The combination of preoperative discontinuation of an anticoagulant, oral steroid use, and perioperative-specific risk factors likely contributed to the early recurrence of embolism. Particular caution is warranted when selecting postoperative anticoagulants, there is a history of discontinuing anticoagulants, or the patient is taking medications associated with a risk of thrombosis. As described above, the ANAFIE Registry [8] demonstrated that patients receiving DOACs had lower rates of thromboembolic events than those receiving warfarin. Notably, among the 8233 patients on warfarin enrolled in this trial, the median time in therapeutic range was not markedly high. This study evaluated the incidence of embolic events among outpatients on anticoagulant therapy, and there were likely to be intervals during which the optimal therapeutic range was not continuously sustained. Therefore, during hospitalization, where strict dose adjustment and maintenance of the optimal therapeutic range are possible, as in the immediate postoperative period, selecting warfarin may sometimes be effective. One approach for the early postoperative period, when the risk of thromboembolic recurrence is high, is to select warfarin for its ability to allow strict dose adjustment. Treatment could begin with warfarin plus heparin for several days, followed by continued warfarin under strict monitoring. However, no evidence supports the effectiveness of switching to warfarin, and in fact, some reports suggest that continuing DOAC therapy is preferable. According to a meta‑analysis on anticoagulant selection after ischemic stroke [15], switching to warfarin, rather than continuing the same DOAC or adjusting its dosage, might increase the risk of recurrent cerebral infarction and intracranial hemorrhage. In the present case, the patient developed recurrent embolism after switching to another DOAC, and ultimately changing to warfarin proved effective. Nevertheless, it cannot be ruled out that a similar embolic event might have occurred even if warfarin had been initiated after the first embolic episode. This case suggests that switching to warfarin can be effective in certain situations. As there are few large‑scale comparative studies regarding the selection of anticoagulant therapy after embolic events, further investigation is warranted.

## Conclusions

We report a case of an older woman who developed cardiogenic cerebral embolism during the perioperative period of acute lower limb arterial occlusion due to thromboembolism. Heightened vigilance for recurrent embolic events is warranted in patients with AF who exhibit a high CHA₂DS₂‑VASc score. Although the optimal anticoagulation strategy following such events remains under debate, transitioning to warfarin may, in selected cases, represent a potentially effective alternative.

## References

[1] Doita T, Kikuchi S, Tamaru Y (2025). Clinical features of acute on chronic lower limb ischemia and the importance of underlying arterial disease for revascularization. Circ Rep.

[2] Andraska EA, Phillips AR, Reitz KM (2022). Young patients without prior vascular disease are at increased risk of limb loss and reintervention after acute limb ischemia. J Vasc Surg.

[3] Ohya Y, Matsuo R, Sato N (2022). Causes of ischemic stroke in young adults versus non-young adults: a multicenter hospital-based observational study. PLoS One.

[4] Kirchhof P, Benussi S, Kotecha D (2016). 2016 ESC guidelines for the management of atrial fibrillation developed in collaboration with EACTS. Eur Heart J.

[5] Camm AJ, Lip GY, De Caterina R (2012). 2012 focused update of the ESC guidelines for the management of atrial fibrillation: an update of the 2010 ESC guidelines for the management of atrial fibrillation. Developed with the special contribution of the European Heart Rhythm Association. Eur Heart J.

[6] Patel MR, Mahaffey KW, Garg J (2011). Rivaroxaban versus warfarin in nonvalvular atrial fibrillation. N Engl J Med.

[7] Giugliano RP, Ruff CT, Braunwald E (2013). Edoxaban versus warfarin in patients with atrial fibrillation. N Engl J Med.

[8] Suzuki S, Yamashita T, Akao M (2024). Patient outcomes in very elderly patients with non-valvular atrial fibrillation ― ANAFIE registry ―. Circ Rep.

[9] Sairaku A, Kimura Y, Nakano Y (2025). Clinical outcomes of off-label DOAC underdosing in Japanese patients with atrial fibrillation: a systematic review and meta-analysis. J Thromb Thrombolysis.

[10] Paciaroni M, Caso V, Agnelli G (2022). Recurrent ischemic stroke and bleeding in patients with atrial fibrillation who suffered an acute stroke while on treatment with nonvitamin K antagonist oral anticoagulants: the RENO-EXTEND study. Stroke.

[11] Paciaroni M, Agnelli G, Falocci N (2015). Early recurrence and cerebral bleeding in patients with acute ischemic stroke and atrial fibrillation: effect of anticoagulation and its timing: the RAF study. Stroke.

[12] Brotman DJ, Girod JP, Posch A (2006). Effects of short-term glucocorticoids on hemostatic factors in healthy volunteers. Thromb Res.

[13] Jung F, Pindur G, Kiesewetter H (1992). Plasma viscosity dependence on proteins and lipoproteins: results of the AACHEN study. Clin Hemorheol Microcirc.

[14] Johannesdottir SA, Horváth-Puhó E, Dekkers OM (2013). Use of glucocorticoids and risk of venous thromboembolism: a nationwide population-based case-control study. JAMA Intern Med.

[15] Romoli M, Paciaroni M, Marrone N (2025). Anticoagulation strategies following breakthrough ischemic stroke while on direct anticoagulants: a meta-analysis. Neurology.

